# Advances in clinical staging, early intervention, and the prevention of psychosis

**DOI:** 10.12688/f1000research.20346.1

**Published:** 2019-11-29

**Authors:** Tina Gupta, Vijay A. Mittal

**Affiliations:** 1 Psychology, Northwestern University, 2029 Sheridan Road, Evanston, IL, 60208, USA; 2 Department of Psychiatry, Northwestern University, 420 E. Superior Street, Chicago, IL, 60611, USA; 3 Institute for Policy Research, Northwestern University, 2040 Sheridan Road, Evanston, IL, 60208, USA; 4 Department of Medical Social Sciences, Northwestern University, 420 E. Superior Street, Chicago, IL, 60611, USA; 5 Institute for Innovations in Developmental Sciences, Northwestern University, 633 N. St. Claire Street, Chicago, IL, 60611, USA

**Keywords:** Psychosis Risk Syndromes; Psychosis; Clinical Staging; Intervention; Prevention

## Abstract

The development of effective intervention and prevention strategies among individuals with psychosis risk syndromes may help to reduce symptomatology and conversion to a psychotic disorder. Although strides have been made in this area, more work is needed, particularly given the setbacks that remain (such as heterogeneity among this group). There has been a shift with the introduction of clinical staging models toward expanding current intervention and prevention efforts to a more developmental and transdiagnostic approach. With this, this article seeks to review treatments both recently and currently discussed in the staging literature, introduce advances in psychosis risk syndrome treatments that may be beneficial to consider in clinical staging heuristics, and pinpoint other promising options.

## Introduction

Psychotic disorders such as schizophrenia typically emerge early in adulthood, during a time when adolescents and young adults are beginning to gain a sense of independence and develop skills intended to set them up for life ^1^. These disorders are common, chronic, and characterized by a variety of symptoms (for example, hallucinations and delusions) that can impact functional outcomes and overall quality of life ^2^. To date, intervention efforts for psychotic disorders have shown promise, although challenges (for example, costs, side effects, availability, and non-adherence) remain.

As a result, in the past decade, there has been a push to intervene prior to psychosis, before symptoms impacting motivation or pleasure and factors such as illicit drug dependence or impacted cognition get in the way of optimal intervention efficacy. Evidence suggests considerable promise for an early treatment approach in lowering symptoms in the high-risk period ^3– 8^ and there is hope that these approaches may eventually allow us to stop psychosis from progressing entirely (or lessen severity and improve course if onset does occur) ^9^. However, many of the same challenges seen in schizophrenia have carried over to this prodromal period. Furthermore, several factors such as heterogeneity of clinical symptoms and trajectories as well as highly prevalent comorbidities (for example, social anxiety and mood disorders) add further complications ^10^. In addition, administration of these interventions takes place in an ever-shifting context in which clinicians are tasked with balancing often-competing goals—including a desire to limit unnecessary treatments (only one to three out of 10 persons at high risk will go on to develop a formal psychotic disorder and so the most costly invasive and stigmatizing treatments are not often warranted) ^11, 12^—with providing the most effective treatments for those who are indeed most likely to transition. Furthermore, clinicians are faced with the priority to address the needs of help seekers; regardless of outcome, most individuals at high risk for psychosis are seeking help for a range of pressing psychiatric symptoms.

One promising area that has the potential to address challenges in developing effective prevention and intervention approaches during risk states is the clinical staging model. Clinical staging recognizes that psychotic disorders emerge over time in the course of an individual’s life and across different development stages. The staging framework can serve as a roadmap for intervention, allowing the ability to target specific, developmentally relevant risk markers and resulting in the possibility to delay or prevent psychosis symptom onset, broader psychopathology, and the implementation of safer intervention strategies (for example, fewer side effects) earlier on in symptom course ^13– 15^. Within this developmental staging context, this article aims to discuss existing (first-generation) interventions and also introduce the most cutting-edge and promising new treatments; highlight the etiology of psychosis, potential mechanisms, and complicating factors; and pinpoint future directions.

## Psychosis risk syndromes

### Emergence and characterization

Adolescents and young adults who fall under the “high-risk state” designation are most often classified with a psychosis risk syndrome (commonly termed an ultra or clinical high-risk or prodromal syndrome) when they show recently emerging (or worsening if more long-standing) attenuated psychosis symptoms (hearing vague whispers, seeing fleeting shadows, feeling confused about what is real, reading into coincidences, and detecting a “special” meaning in mundane events/experiences) as well as functional decline in school/work and social relationships ^1, 16, 17^. Other valuable conceptions of psychosis risk syndromes have focused on brief intermittent psychotic symptoms in which there is the presence of brief (less than 1 week) transient psychotic symptoms, trait risk factors such as genetic vulnerability ^1^, and basic symptoms that are subclinical, subtle, and self-experienced (that is, private and apparent only to the individual) disturbances in aspects of functioning, including affect, speech, and body perception ^18– 20^. As noted, whereas some individuals meeting criteria for a psychosis risk syndrome may go on to develop psychotic disorders, a larger percentage do not (for example, instead remaining at the same symptom level or even improve), which together may provide critical insights and clues regarding the pathogenesis of psychosis more generally ^9, 21^. For those 10 to 30% who do transition, the prodromal period (typically lasting 2 years prior to onset) is characterized by increasing frequency and severity coupled with elevating distress, related impairment, impacted insight, and fuller conviction (that is, a symptom cannot be explained away by imagination) ^16, 22^. Other symptom characteristics of this group include negative symptoms (for example, social anhedonia and avolition), thought disorder, motor signs, and decline in cognitive functioning (for example, deficits in working memory and social cognition) ^1, 6, 16, 23^. Furthermore, changes in brain health (for example, abnormalities in neuromaturational processes) have been widely identified within the literature ^24– 26^. Notably, there is consensus in both research and practice that the clinical presentation and needs of these individuals differ from those with multiple chronic episodes ^27^.

Generally, the goals for interventions targeting psychosis risk syndromes are to (1) limit the duration of untreated time by promoting early detection, (2) treat psychosis risk symptoms and characteristics (positive and negative symptoms, cognitive decline, emotional dysfunction, and emerging brain dysfunction) contributing to disability, (3) address auxiliary clinical symptoms and disorders, (4) provide psychoeducation around maladaptive self-medication behaviors (for example, substance abuse), (5) delay and prevent the onset of psychosis altogether and lessen symptom severity of cases that do transition ^1, 16^, and (6) inoculate caretakers and put resources in place prior to what may be an impending stressful and tumultuous time.

### Caveats

When considering treatment for psychosis risk syndromes, clinicians should consider several important points. First, the etiology of psychosis is multifaceted, often with several pathways and causes, making the identification of treatments challenging ^28^. On the other hand, single risk factors (for example, childhood abuse) can have a pluripotent effect and lead to numerous outcomes, including psychosis ^1, 16, 17^. Another challenge is the heterogeneous nature of this group ^10^; that is, each individual within this category presents with a unique clinical presentation, highlighting that individuals with different symptoms may vary in the way they are receptive to, and how they benefit from, intervention approaches ^29^. This includes the fact that most individuals who meet criteria for a psychosis risk syndrome also exhibit symptoms or carry formal clinical diagnoses of other forms of serious mental illness, including anxiety, mood disorders, substance abuse, attention-deficit/hyperactivity disorder, post-traumatic stress disorder, and autism spectrum disorders ^22^. Relatedly, recent evidence of biologically distinct subgroups in the psychosis risk period shows a unique course as well as distinct brain vulnerability ^30^. This highlights that although traditional “one size fits all” approaches may not be optimal, there is tremendous possibility here. Indeed, an individualized medicine approach may be possible in the near future, where distinct patterns of symptoms and vulnerability signs may warrant one treatment over another ^31^. As noted above, not all individuals with a psychosis risk syndrome will transition, but even so, this group requires intervention as well (for example, opportunities for intervention include reduction in symptom severity) ^31^. Finally, it is imperative to consider that individual differences as well as cultural, ethnic, and racial factors influence the assessment and experiences of psychosis risk syndrome and that, although these are currently understudied ^32^, they should remain a central consideration of any treatment.

### First-generation interventions

Historically, most treatments for psychosis risk syndromes have focused primarily on the individual and, over time, have begun to incorporate the family unit ^33^, particularly given evidence suggesting that family environment can predict symptomatology ^34^. However, there is still a limited focus on broader systems such as schools and communities, which play a critical developmental role in symptom onset. Furthermore, most of the previous literature has focused on psychosis risk syndromes and has not included earlier developmental stages such as perinatal and premorbid (infant to late childhood) periods; as a result, we may be missing critical opportunities to intervene. Additionally, although prevention and intervention strategies show promise in psychosis risk groups, we are still working toward gold-standard treatments and this is due in part to the numerous caveats noted earlier (for example, failure to account for subgroups) and reports of inefficacy in interventions within the current literature to treat both positive and negative symptoms ^35, 36^. In this section, we discuss several examples of what we refer to as first-generation interventions (rather than “established” interventions), highlighting the historical and ongoing obstacles in finding effective treatment options for those with psychosis risk syndromes ( Table 1).

**Table 1.** Past, present, and future intervention and prevention strategies for psychosis risk states.

First-generation	Recent advancements	Promising future directions
• Low-dose antipsychotic medications • Other medications (for example, selective serotonin reuptake inhibitors) • Cognitive behavioral therapy • Family-based interventions • Polyunsaturated fatty acids • Cognitive remediation	• Exercise • Smartphone applications • Virtual reality • Brain stimulation	• Social media • Biofeedback • Oxytocin-based interventions • Mindfulness-based interventions • Sleep hygiene • Enhancing protective factors • Eliminating barriers to care • Reducing stigma • Mental health prevention in urban planning

First-generation medications are what we refer to as established treatments historically used in the treatment of psychosis risk states.

Several options for treatment in psychosis risk have been directly informed by interventions proven to be effective in formally psychotic patients, and, generally speaking, each has a strong mechanistically driven goal. Currently, antipsychotic medications are the cornerstone treatment for individuals with psychotic disorders, targeting and decreasing positive symptoms (for example, hallucinations) ^37^. In psychosis risk syndrome groups, there is some evidence for the efficacy of antipsychotic medication at low doses to treat positive symptoms ^38^. However, given common side effects, non-adherence, and expense ^37^, other treatments may be more effective initially given the risk-benefit profile of these medications ^39^. Other experts (for example, the International Early Psychosis Association Writing Group) have argued that these medications should never be considered for the psychosis risk syndrome. It should be noted that several lines of evidence support efficacy of other psychotropic medications (for example, selective serotonin reuptake inhibitors/serotonin–norepinephrine reuptake inhibitors) for targeting commonly occurring affective symptoms. Of course, this is a complex area that requires significant expertise as inappropriate medication selection can trigger manic episodes (bipolar disorder is a common comorbid condition in psychosis risk individuals) or potentially exacerbate the course of psychosis (for example, if stimulants are inappropriately prescribed when negative symptoms are mistaken for attention deficits).

Fortunately, investigators have also explored other viable options. For example, cognitive behavioral therapy (CBT) is considered one of the most commonly employed psychosocial treatments for psychosis risk syndromes ^29^. CBT is evidenced to be effective and involves restructuring thoughts and changing behaviors that maintain symptomatology and levels of distress, and studies have found that CBT can reduce symptomatology and risk of conversion to psychosis ^1, 40– 42^ as well as comorbid symptoms such as anxiety and depression ^22^. However, limitations of work in this area continue to include small sample sizes, high dropout rates, failed replication studies, and inconclusive results.

Psychosocial interventions have shifted to provide intervention to not only the individual but also the larger family system, and growing research indicates the importance of a healthy family environment in improving psychosis risk symptomatology ^33, 34^. For example, some work suggests the efficacy of CBT intervention in which family members are taught CBT in order to apply these tools and techniques in the home environment, and evidence shows decreases in offspring symptoms (for example, positive, negative, and depression) ^43^. Furthermore, other promising family-oriented interventions have been developed to increase support and communication within the family system and reduce stigma ^33, 44^. However, as with psychopharmacological approaches, there remain challenges with sample size, single-group designs, and resource demands in research and practice.

In addition to CBT and other family-based interventions recommended as a first line of treatment, long-chain omega-3 polyunsaturated fatty acids (PUFAs) have been used as an intervention, particularly in early stages, and this area continues to grow ^45, 46^. Some evidence suggests that PUFAs may reduce risk of progression to psychosis ^47^, whereas other evidence indicates no evidence of efficacy ^46^.

Furthermore, there is established evidence that cognitive deficits are characteristic of individuals with psychosis risk syndromes, including impairments such as working memory and social cognition ^23, 25, 46^. Given the detrimental impact that abnormalities in cognition can have on an individual, thus impacting aspects of functioning ^28^, such as the ability to maintain a job or perform well in school, finding ways to improve cognitive function in these groups is imperative ^48^. One avenue to improve cognitive function in this group is cognitive remediation; these interventions involve specific training of social and cognitive functions and continue to show promise in the field ^49, 50^.

Together, although these interventions approaches are promising ^42^, challenges and setbacks remain. Similarly, in the largest intervention meta-analysis to date examining the effects of specific treatments in psychosis risk states, 14 studies were examined (for example, examining treatments such as antipsychotic medications, CBT, and family therapy), and findings indicate no robust evidence in favor of any intervention for improving positive symptoms ^35^, and similar conclusions were drawn from other meta-analytic evidence examining interventions for negative symptoms ^36^. This suggests that continued work is warranted in order to develop and implement efficacious targeted treatments in psychosis risk syndrome groups.

### Clinical staging

As previously described, many of the first-generation interventions have focused on psychosis risk syndromes specifically. However, challenges in progress are due to issues such as a large focus on conversion to psychosis, which is problematic given that many individuals with psychosis risk syndromes do not convert but still exhibit psychopathology/worsening of symptoms. Similarly, few people who go on to develop psychosis have not accessed mental health services related to symptoms prior to onset ^51^, and there may be concerns regarding potential stigma and limited resources. Additional limitations are observed in the literature and include inconclusive results and methodological issues (sample size and single-group designs). As noted, interventions have focused on the psychosis risk period, which is critical; however, there are other developmental periods (for example, prenatal and premorbid) that may provide an opportunity for intervention, which may have more long-lasting impacts, delaying or preventing psychosis onset well before there is functional impact, overall having the potential to alter the path to future psychosis. As such, from this developmental perspective, the field has made a shift toward a new model that can inform intervention strategies, taking into account developmental and transdiagnostic information ^14^. Specifically, increasing epidemiological evidence indicates that mental illness develops over time, across different stages of disease progress, and this is the notion underlying clinical staging ^13, 14^, which will be discussed in this section.

Clinical staging refers to the idea that the development of mental health challenges has the potential to progress to formal illness at some point in an individual’s life and is one possible avenue that extends the focus on not only psychosis risk syndromes but also earlier stages within the course of illness. The introduction of clinical staging into psychiatry has been instrumental in setting up a blueprint for the development of prevention and intervention strategies among severe mental illness ^13– 15^. Critically, staging models are important in that they define risk markers and interventions can be offered on a broader level earlier in treatment, resulting in less harmful, expensive, and stigmatizing interventions initially and addressing concerns regarding predictive power. This approach provides intervention options not only for those solely considered at imminent risk for psychosis but also for low-risk groups such as those that report mild levels of anxiety and depression or learning difficulties; this trend may help to overcome the “prevention paradox” ^52, 53^, a phenomenon that, until now, has been hindering treatment advancements. In earlier stages, some individuals may not progress to psychosis, but if they do, more specialized treatments later in disease progression are available and deemed warranted ^15^.

In clinical staging models, illness transcends the boundaries of diagnostic criteria, emphasizing instead where in a developmental trajectory (through stages) an individual may lie ^15^. Importantly, underlying these stages and the movement to the next level are risk factors (for example, biological, social, and environmental) that are linked specifically to psychosis or may be more representative of broader psychopathology, and staging allows the possibility for this differentiation ^14^. Mapping risk markers on a clinical staging heuristic is helpful in that we can consider at what stage an individual is showing earlier signs of psychosis risk, intervening with less invasive approaches at that specific point, prior to rapid disease progression. To be more specific, from a psychosis risk state perspective, stage 0 refers to the prenatal period and incorporates prenatal health; markers include prenatal infections and complications ^53– 57^ and familial risk ^16, 58, 59^. Next, stage 1a includes mild symptoms (that may be more representative of a premorbid period) commonly emerging in childhood and preadolescence ^60^. Signs/symptoms that someone is in this stage include low levels of anxiety and depression, impairments in functioning, or mild cognitive and motor issues or a combination of these. Risk factors in this stage include low socioeconomic status, poor coping, refugee status, migration, neighborhood-level social deprivation, urbanicity, cannabis exposure, and stress ^61– 63^, and biomarkers include neuroinflammation such as increased interleukin-6 (IL-6) levels ^64^. Here, some of the first-generation treatments such as CBT might be employed, although most of those discussed are designed for the next stage. Stage 1b signals moderate psychosis risk signs/symptoms typically occurring in adolescence and early adulthood. Signs/symptoms include sleep dysfunction, cognitive decline, social isolation, challenges in work and school environments, unusual thoughts, and perceptual abnormalities. Many of the risk factors from 1a carry over but may intensify (for example, more directed bullying and peer isolation), and new patterns may also emerge (self-medication and substance abuse). Vulnerability markers include a range of factors, including biological signs such as GABAergic abnormalities ^25^, neuroinflammation ^64, 65^, and hypothalamic–pituitary–adrenal (HPA) axis dysfunction ^66^ as well as behaviors such as difficulty coping ^67^ and substance use ^68^. Stages 2 to 4 cover the first psychotic episode through the chronic psychosis ^13, 14, 69^ and are outside the scope of this review.

An important point is that some risk factors are likely to signal a more broad and general risk for psychopathology whereas other markers such as motor abnormalities and negative symptoms have been found to be specific to psychosis, and clinical staging has the potential to allow for process- and symptom-oriented intervention (for example, general screening for other psychopathology such as depression and anxiety and then more specialized treatments to narrow assessments in later stages).

## Recent advances in prevention and intervention

The field is making a shift in understanding risk markers to a new staging model perspective, in which course of illness is viewed along a developmental and transdiagnostic path. As a result, efforts have been made to identify types of interventions that may be useful at different points on this trajectory; earlier treatments involve more general intervention, and later stages require more personalized approaches. While work is still growing in this area, this conversation has the potential to identify and prevent psychosis onset earlier on, even prior to symptom severity and functional impairment ^14^.

**Figure 1.**
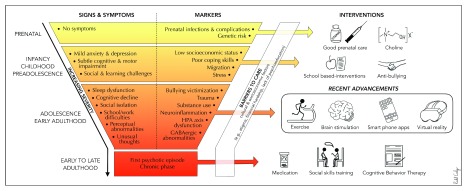
Signs, symptoms, markers, and interventions from a clinical staging perspective in psychosis risk syndromes. Many of the signs and markers may occur in different stages and overlap between stages. Furthermore, it is important to note that many of the individuals who may exhibit signs and symptoms that put them on the trajectory toward developing a psychotic disorder may not go on to develop a diagnosis and instead may improve or develop a different psychopathology. HPA, hypothalamic–pituitary–adrenal.

The remainder of the article seeks to review treatments, both recently or currently discussed in the staging literature (for example, choline and school-based interventions) ^70^, and other novel and promising options (for example, exercise, smartphone applications, virtual reality [VR], and brain stimulation) in order to introduce the inclusion of these treatments in clinical staging heuristics ( Figure 1). First, we highlight early developmental stages in the hope that the field will continue to move in this important direction (spanning stages 0 to 1a). Then, we discuss newer interventions during the adolescence and young adulthood stage, also categorized as the psychosis risk period, which is the primary focus of this article.

### Prenatal period: prenatal care and choline

Opportunities for treatment during the prenatal period have been discussed in clinical staging paradigms ^1^. These treatments stem from a long-standing body of literature suggesting that maternal health during fetal offspring development is important in the prevention of offspring psychosis ^71, 72^. There is evidence of risk for psychosis in studies finding links with offspring born during the winter/spring ^57^, complications during birth ^58^, maternal nutritional deficiency ^71^, and infection during pregnancy ^54– 57^; these data have been instrumental and suggest that early environmental insults may impact infant brain development and increased likelihood of psychosis ^70^. As a result, intervention approaches have traditionally involved psychoeducation with an emphasis on ensuring maternal nutrition and proper immunization; however, specific prenatal preventative intervention work has been more limited ^72^. Growing research indicates that choline-enhancing diets (which may decrease effects of mother’s infections on fetal brain development) for pregnant women may be a means to protect against neural and cognitive deficits in offspring ^73, 74^. For example, Freedman and Ross ^72^ (2017) administered a double-blind, placebo-controlled trial in which 100 women were administered phosphatidylcholine supplementation in the second trimester of pregnancy. Babies with perinatal supplementation showed more cortical inhibition and went on to have fewer attention symptoms and less social isolation compared with the placebo-treated babies. While this area of research continues to grow, there is promise, particularly if research paradigms can be extended outside of high-income countries to examine the generalizability of current findings ^75^.

### Preadolescence and childhood: school-based interventions

School-based interventions have also been proposed in clinical staging models ^1^. The etiology of these interventions comes from the conceptualization of psychotic disorders such as schizophrenia from a diathesis stress model indicating that the interaction between biology (for example, risk from a first-degree relative with psychosis) and environment (for example, stress) can “set off” psychosis onset ^76^. Research identifying stress as a risk marker is not new to the field, and additional components of “stress” now include social factors that tend to emerge in school environments and within peer social circles, such as bullying victimization ^77^.

Given that school settings form a strong basis for childhood development (for example, social skills and coping with stress), targeting school settings is of value. As a result, a potential intervention avenue is providing resources and psychoeducation to educators that can aid in the implementation of school-based interventions. Similarly, there has been an emphasis on educating school-based providers in the detection and screening of psychosis risk; however, there are reports that school-based providers rate themselves as less confident for treating psychosis compared with other mental health difficulties, suggesting that further training in these settings is warranted ^78^. Development of interventions to mitigate or prevent this stress is ongoing, and findings in which links have been detected between environmental stress and conversion to psychosis continue to form as a foundation of this work in psychosis risk syndrome groups ^29, 70^. Along these lines, interest in the implementation of anti-bullying programs has recently gained attention in response to accumulating evidence suggesting positive relationships between bullying and early psychosis symptomatology ^79– 82^. Although work is still needed in order to test the efficacy of anti-bullying in psychosis risk prodromes, evidence in healthy groups shows potential for this area. For example, Nocentini and Menesini (2016) applied an anti-bullying program in Italy in a randomized controlled trial of students in grades four and six (N = 2042) ^83^. Students were randomly assigned to the intervention or a control condition; findings revealed that the intervention reduced bullying and victimization. Furthermore, a study of 264 fourth-grade children with elevated levels of aggression found that implementation of an anti-bullying program reduced aggressive behaviors ^83^. Anti-bullying programs have the potential to aid in preventative efforts by providing psychoeducation to what bullying is and the impacts of these behaviors on other peers.

### Adolescence and early adulthood

Work investigating treatments for stages focused on maternal health and childhood/preadolescence continues to grow and has been of interest and many of these interventions have been conceptualized and placed on the clinical staging heuristic. In the next clinical stages, in which individuals may be characterized by a psychosis risk syndrome, research on prevention strategies is rich, and there are several promising avenues. One important takeaway is the importance of including these interventions within a clinical staging heuristic, particularly because each of these strategies can be viewed as developmentally specific, falling within the adolescent and early adulthood period. The rest of this article seeks to discuss newer treatments that may be beneficial for this specific age group and developmental period.

### Exercise

Increasing evidence indicates that regular exercise can decrease symptomatology and improve brain health in individuals with mental health disorders, including schizophrenia ^84, 85^. Given the high rates of obesity (often a side effect of antipsychotic medication use leading to additional psychological and physical health problems) among psychosis groups ^86, 87^, exercise may be an affordable, effective option, targeting symptoms and markers that may begin to develop during adolescence and early adulthood. Given the favorable outcomes observed in those with formal psychosis ^84, 88^, exercise may be a useful prevention and intervention strategy prior to psychosis onset.

In psychosis risk syndromes, previous studies indicate that these individuals spend significantly more time engaging in sedentary lifestyles than healthy groups do ^83– 85^. Furthermore, there is evidence that when individuals with psychosis risk syndromes do engage in exercise, they often participate in activities that require minimal social interaction ^89^. Similarly, there are findings indicating that these risk groups engage in less indoor/outdoor activities and strength/flexibility training and have more barriers to exercise such as motivation ^90– 92^. In one study using a sample of 51 individuals with psychosis risk syndromes and 37 controls, greater symptomatology was associated with lower self-perception related to engaging in physical activity (for example, lack of skills to do certain types of exercises) and motivation to exercise ^91^. These studies provide increasing evidence that highlights atypical behavioral patterns, shedding light on our conceptualization of the etiology of psychosis. Furthermore, these data provide a framework for the implementation of exercise interventions among psychosis risk syndrome groups. 

As noted above, exercise interventions have been shown to decrease symptomatology in psychosis populations ^84, 88, 93^, and although work in implementing exercise interventions in psychosis risk syndrome groups is more limited ^90, 94^, it continues to grow, and preliminary findings indicate tremendous promise in this area. For example, our group employed a pilot study in which nine psychosis risk individuals engaged in 12 weeks of exercise two or three times a week and exercised between 65 and 85% of maximal oxygen capacity for 30 minutes per session. Findings revealed decreases in symptomatology and increased social and role functioning, neurocognitive performance, cardiovascular fitness, and functional connectivity between the left hippocampus and occipital cortex post exercise ^94^. Based off of the success of this feasibility study, our group has moved to a second phase of this study to continue to examine the effectiveness of cardiovascular exercise in promoting brain health (for example, medial temporal health) and improving related symptoms (for example, hearing whispers, feeling emotionally detached from others, cognitive difficulties, and everyday functioning).

### Smartphone applications

The advancement and integration of systems such as smartphone applications (or apps) into our society have become increasingly common among individuals with mental health difficulties and in mental health care in general. This is particularly relevant in adolescence and young adulthood, which is commonly the age range in which individuals are introduced to smartphone apps ^95, 96^. The use of smartphone apps may be an intervention option for adolescents and young adults, particularly given that these modalities are becoming increasingly used and enjoyed. As such, smartphone apps may exhibit higher adherence rates among psychosis risk syndromes and may be an assessment and intervention tool that can be accessed anywhere and at any time.

For many individuals, the use of smartphone apps comes with barriers ^97^, such as socioeconomic factors, and, of course, these tools have potential hazards, such as distraction from engaging with in-person relationships, internet addiction, and fostering stigma. Even so, there is evidence that individuals with mental health challenges do own smartphones ^98^, and the implementation of smartphone apps has been found to be possible in early psychosis to monitor symptoms, mood, and sleep difficulties in conjunction with clinical care (clinicians can review these ratings in treatment sessions and help develop more targeted weekly interventions) ^99, 100^. The benefit of these tools is that they can provide real-time intervention, and given the rates at which individuals own smartphones and enjoy using them, the likelihood of treatment adherence may be high ^101^; however, more work in this area is needed before definitive conclusions can be made. Furthermore, according to the National Institute of Mental Health, these tools have other advantages such as being convenient, low in cost (for example, some are even free), and available at any time and serving as an introduction to other types of care. Importantly, these tools may have the ability to foster international collaborations given the increasingly used and accessed nature of smartphone apps ^102^.

Studies examining the use of smartphones apps are under way, and several registered clinical trials are collecting data testing the use of specific apps for interventions (for example, aimed at educating how to recognize symptoms, conducting mindfulness practices, and learning problem-solving skills) in psychosis and risk syndrome populations ^103, 104^. Furthermore, questions regarding the feasibility of using these apps remain, and as a result, studies are working toward examining whether it is possible to implement these tools in risk populations ^99, 105^. There is some evidence already in early psychosis for the efficacy of using smartphone apps in conjunction with other modalities (such as CBT) to monitor symptoms, to complete intervention modules (for example, thought records) ^105^, and to use as an add-on treatment tool as a means to administer weekly and daily surveys to supplement therapy visits ^94^.

### Virtual reality

Within the literature, social and role deficits among psychosis risk groups are commonly identified ^1, 16, 106, 107^, and there is evidence that psychosis risk groups have fewer social relationships and report feeling lonelier ^108^. Furthermore, findings from a prospective, longitudinal study through assessing age-related changes in social and role functioning suggest that while there were no changes over time in healthy controls in social and role functioning, deficits in social and role functioning were observed as early as age 12 in psychosis risk individuals that later converted. Additionally, this pattern continued over time, with some improvements in different stages, but overall lagging behind healthy controls and individuals with psychosis risk syndromes that went on to not convert ^109^. These data highlight the need to continue to develop ways to improve social and role functioning among this group, particularly given that these impairments are predictors of increased symptomatology and conversion to psychosis ^1^. To address these issues, VR may be one way to increase functional outcome.

The ability to simulate interactive situations with VR can allow for assessment of real-life responses to environmental stress and can also be used as an intervention strategy. VR has been introduced into clinical psychology as a means of exposure-based treatment, showing efficacy in application and improvement in symptoms in populations with anxiety disorders ^95^. VR has been used in order to better understand mechanisms in psychosis ^110^. An example of using VR to inform our understanding of psychosis and risk stems from a study in which Veling *et al*. (2016) ^111^ used VR to assess environmental responses in social situations. Specifically, samples of individuals with psychosis, psychosis risk syndromes, and familial risk and healthy controls used VR in which a bar scene was simulated and participants were walking into the bar with different levels of environmental social stress. Findings indicated that paranoia and subjective distress increased in parallel with the amount of environmental social stress. Similarly, in addition to using VR as a means to research symptomatology, clinicians can use VR as a tool for intervention. For example, with computer-generated interactive environments, individuals can learn tools and techniques to combat challenging situations such as interpersonal conflicts (for example, bullying and social exclusion), improving both social skills and resiliency ^95, 110^. Recently within the literature, VR has been used predominately in psychosis populations ^112^. Additionally, VR has been used in order to encourage individuals with psychosis experiencing persecutory delusions to test these beliefs ^89^.

### Brain stimulation

Transcranial direct current stimulation (tDCS) may be a promising intervention tool when symptomatology and cognitive function begin to be impaired ^113– 117^. This technique has been found to reduce symptomatology ^118, 119^ and improve neurocognitive functions ^120^ and social cognition ^117^. While work in this area is still under way in order to answer remaining questions such as side effects, appropriate length of stimulation, and overall efficacy, this technique has potential to serve as a prevention and intervention strategy, particularly when symptoms begin to emerge.

This non-invasive tool works by modulating cortical excitability by releasing a weak electrical current (2 mA or less) between two electrodes that are placed on the scalp ^115^. Various montages, such as bilateral placements (placing a positively charged anode electrode over a region to increase excitability and a negatively charged cathode over an irrelevant region to decrease excitability), can be used with this technique. Furthermore, sham (placebo) conditions have been applied where participants may receive stimulation briefly (typically for 30 s) while other parameters remain constant ^114, 116, 121^.

Although many of the tDCS studies have been conducted in schizophrenia ^113, 114, 116, 117^, our group applied tDCS to individuals reporting high levels of non-clinical psychosis (NCP) (individuals who are non-help-seeking and exhibit infrequent symptoms on the lower end of the psychosis continuum), an analogue group to psychosis risk syndromes. In this study, we found that tDCS improved procedural learning performance (normalizing performance to the level of controls) when compared with controls ^118^ after being in the active stimulation condition. These data suggest that tDCS may be useful in other groups on the psychosis continuum and may be a useful treatment option, but further work is needed. Furthermore, this study highlighted the potential to use NCP as a safe analogue population, which may be useful in the development of newer treatment interventions for psychosis risk syndrome groups. Although work in this area is still growing, tDCS may be a tool that could be applied when early signs of symptom progression and cognitive decline are observed and begin to interfere with daily life. Other neuromodulation techniques that continue to be understood and may hold potential as prevention and intervention tools include transcranial alternating current stimulation (tACS) ^122^ and repeated transcranial magnetic stimulation (rTMS) ^123, 124^ and these techniques in conjunction with other traditional interventions such as cognitive remediation ^125^.

## Additional considerations and future directions

Intervention and prevention research continue to make important advancements in the field with the goal of reducing symptomatology and the likelihood of psychosis onset. Although traditional treatment approaches and newer advances in the prevention of psychosis hold promise, developments are still warranted, and the use of clinical staging models can help provide direction. 

As the field continues to develop efficacious prevention and intervention strategies, it is important to consider challenges that remain. For example, each individual presents with a unique clinical presentation (for example, differing symptom and cognitive scores and level of intact social and role functioning), which can complicate implementing an appropriate intervention. With this, monitoring symptoms throughout the course of treatment is critical and moving toward more personalized approaches is of value. Critically, given the heterogeneous nature of individuals with psychosis risk syndromes ^10, 126^, clinical staging has the potential to inform these strategies ^15, 127^. A direction that may provide further advancements in treatment is further research of specific developmental periods such as prenatal and premorbid stages and relevant risk markers and signs. There may also be utility in moving beyond an approach that examines conversion to psychosis to one that emphasizes other functional outcome measures such as education, employment, and social interactions ^128, 129^. Furthermore, efforts to develop prediction calculators based on variables such as demographics, presence of other comorbid psychiatric disorders, neurocognition, and exposure to early life adversity and trauma ^59, 130, 131^ have the potential to help the field hone in on prevention and intervention efforts for specific subgroups ^131^.

The newer advancements in the prevention of psychosis discussed in this article hold promise. However, it is important to note that more work is needed to understand which intervention may be most effective for different developmental illness stages. Additionally, research investigating the efficacy of these interventions, such as the implementation of brain stimulation, is ongoing. Furthermore, there are several other promising advancements currently discussed within the field ( Table 1). For example, another possible intervention is the use of social media, particularly in the effort to improve social functioning, a common deficit observed among psychosis risk individuals ^97^. According to Torous and Keshavan (2016), social media has the potential to provide peer support at any given time and may be a more approachable and accessible means to connect with other individuals ^97^. However, research assessing the impacts of social media on social functioning is limited and warranted. Additionally, further research in understanding additional intervention tools such as biofeedback to treat comorbidity such as anxiety ^132^, oxytocin-based interventions ^35^, and mindfulness-based interventions ^133^ can be useful. Other possible intervention targets include sleep hygiene, particularly given evidence showing links between sleep disturbances and symptomatology ^134^.

Another critical point to consider and build on is how communities and broader society can play a role in contributing to intervention efforts. Avenues for this direction include the consideration of cultural, ethnic, and racial factors previously noted that can potentially be barriers and obstacles to seeking care ^32^. Attention to additional environmental risk factors is critical and can be implemented on the level of urban planning. For example, there are potential preventative strategies to address findings indicating links between urbanicity and psychosis ^135^, such as the consideration of implementing more green space and overall improving of housing infrastructure. Furthermore, stigma associated with mental illness is a challenge to overcome and future work is needed in this area ^136^. Given that psychosis in particular is highly stigmatized, factors can interfere with the likelihood that an individual in need of care seeks it and overall quality of life ^137^. Although this area of research is ongoing, there are findings suggesting efficacy of stigma-related interventions ^138^ and work examining ways that researchers and providers can minimize stigma when working in this area ^12^.

Other future directions include using NCP groups as a safe analogue population to psychosis risk syndromes. This may be useful in the development of new treatment interventions as a means, for example, to assess treatment feasibility. Similarly, there may be important insights obtained from understanding stages beyond psychosis risk syndromes among individuals having brief psychotic disorders but not yet exhibiting formal, chronic psychosis ^139^. Integrating data from other sources such as medical records and registries in order to examine functional outcome may be useful ^140^. Although there is increasing research examining protective factors such as family environment ^34^, psychosocial interventions ^141^ and resiliency ^142^, research into the efficacy of the implementation of treatment approaches to enhance the role of these protective factors is imperative.
